# Mitochondrial Genome Characteristics Reveal Evolution of *Danxiaorchis yangii* and Phylogenetic Relationships

**DOI:** 10.3390/ijms26020562

**Published:** 2025-01-10

**Authors:** Xuedie Liu, Huolin Luo, Zhong-Jian Liu, Bo-Yun Yang

**Affiliations:** 1 School of Life Sciences, Nanchang University, Nanchang 330031, China; liuxuedie1995@126.com (X.L.); 572460991@163.com (H.L.); 2 Key Laboratory of National Forestry and Grassland Administration for Orchid Conservation and Utilization at College of Landscape Architecture and Art, Fujian Agriculture and Forestry University, Fuzhou 350002, China

**Keywords:** *Danxiaorchis yanii*, organelle genomes, phylogenetic analysis, gene selective pressure

## Abstract

*Danxiaorchis yangii* is a fully mycoheterotrophic orchid that lacks both leaves and roots, belonging to the genus *Danxiaorchis* in the subtribe Calypsoinae. In this study, we assembled and annotated its mitochondrial genome (397,867 bp, GC content: 42.70%), identifying 55 genes, including 37 protein-coding genes (PCGs), 16 tRNAs, and 2 rRNAs, and conducted analyses of relative synonymous codon usage (RSCU), repeat sequences, horizontal gene transfers (HGTs), and gene selective pressure (dN/dS). Additionally, we sequenced and assembled its plastome, which has a reduced size of 110,364 bp (GC content: 36.60%), comprising 48 PCGs, 26 tRNAs, and 4 rRNAs. We identified 64 potential chloroplast DNA fragments transferred to the mitogenome. Phylogenomic analysis focusing on 33 mitogenomes, with *Vitis vinifera* as the outgroup, indicated that *D. yangii* is grouped as follows: *D. yangii* + ((*Dendrobium wilsonii* + *Dendrobium wilsonii henanense*) + *Phalaenopsis aphrodite*). Phylogenetic analysis based on 83 plastid PCGs from these species showed that *D. yangii* is grouped as follows: (*D. yangii* + *Pha. aphrodite*) + (*Den. wilsonii* + *Den. henanense*). Gene selective pressure analysis revealed that most mitochondrial and plastid genes in *D. yangii* are under purifying selection, ensuring functional stability, and certain genes may have undergone positive selection or adaptive evolution, reflecting the species’ adaptation to specific ecological environments. Our study provides valuable data on the plastomes and mitogenomes of *D. yangii* and lays the groundwork for future research on genetic variation, evolutionary relationships, and the breeding of orchids.

## 1. Introduction

All eukaryotic cells contain organelles believed to have originated from ancient endosymbiotic events [1]. Plants possess two such organelles: the mitochondrion and the plastid. The mitochondrial and chloroplast genomes (mtDNA and cpDNA) are derived from the genomes of their symbiotic ancestors: an α-proteobacteria and a cyanobacteria, respectively [2]. Plant mitochondrial genomes (mt genomes) have several unique features compared to the conserved plant plastid genomes and compact animal mt genomes. These features include a wide range of genome sizes, multipartite genome arrangements, low gene density, high intron density within genes, trans-splicing associated with different intron groups, RNA-level editing, gene transfer or loss, and foreign sequence capture [3,4,5]. However, sequencing plant mitochondrial genomes is challenging due to the ease of chloroplast DNA contamination during mitochondrial DNA extraction [6], as well as the complex structure of the mitochondrial genome. In addition to the typical circular structure, mitochondrial genomes can also exist in linear, multipartite, branched, and complex configurations. For instance, the mitochondrial genome of *Epipogium roseum* consists entirely of 26 circular chromosomes, while *Gastrodia elata* has 19 subgenomes [7], including 12 circular and 7 linear subgenomes [8].

Although plant mitochondrial genomes represent a valuable source of phylogenetic information, they remain underutilized in phylogenetic studies due to their extreme size variation, low mutation rates, and challenges in assembly [9]. In contrast, chloroplast sequences have been widely used to infer cytoplasmic evolutionary histories, often assuming uniform inheritance patterns [10]. However, mitochondrial and chloroplast genomes in angiosperms do not always adhere to strict maternal inheritance. For example, McCauley et al. (2013) demonstrated paternal leakage in mitochondrial genome inheritance, potentially disrupting the expected linkage equilibrium between cytoplasmic genomes [11]. Such inheritance heterogeneity likely resulted in phylogenetic discordance between mitochondrial and chloroplast genomes. Thus, incorporating mitochondrial phylogenies is crucial for a comprehensive understanding of flowering plant evolution.

Intercellular gene transfers (IGTs) frequently occurred among the nucleus, plastids, and mitochondria, promoting the movement of genetic material within organisms. Evidence of horizontal gene transfer (HGT) involving angiosperm mitogenomes has been documented for decades. Angiosperm mitogenomes often fuse and recombine, facilitating the exchange of genomic segments among mitogenome populations within a single cell [12]. This genetic exchange, combined with the tendency of plant mitogenomes to maintain long intergenic spacer regions, can preserve syntenically intact stretches of foreign-derived sequences over deep evolutionary time. HGT has been shown to occur more frequently between organisms engaged in symbiotic relationships, such as parasitic plants and their hosts [13]. The co-occurrence of both the plastome and mitogenome in plant cells perhaps represents an extreme example of symbiotic intimacy leading to HGT, with plastid-derived sequences being incorporated into the mitogenome early in the evolutionary history of land plants [14].

Orchidaceae is recognized as one of the largest families of angiosperms and is divided into five subfamilies (Apostasioideae, Vanilloideae, Cypripedioideae, Orchidoideae, and Epidendroideae), encompassing approximately 736 genera and 28,000 species [15]. About half of all mycoheterotrophs (~200 spp.) are found within the Orchidaceae family, representing at least 30 independent transitions to mycoheterotrophy, some of which are very ancient [16]. Sequenced plastid genomes of mycoheterotrophic plants can provide useful phylogenetic information and insights into patterns of genome evolution and changes in selection with the loss of photosynthesis [17,18]. Each mycoheterotrophic lineage represents an independent evolutionary experiment on how to survive without sunlight; studying them allows us to infer general vs. lineage-specific correlates of this major evolutionary transition [19]. For instance, fully mycoheterotrophic plants rely entirely on fungi for their carbon supply, leading to the release of selective pressure on photosynthesis-related genes, which are thought to be rapidly pseudogenized or lost in fully mycoheterotrophic taxa.

The genus *Danxiaorchis* [20], placed in the subtribe Calypsoinae (Epidendreae and Epidendroideae), comprises only three recorded species: *Danxiaorchis yangii* [21], *Danxiaorchis singchiana* [22], and *Danxiaorchis mangdangshanensis* [23], all of which lack roots and leaves. In this study, we sequenced, assembled, and annotated the first completed plastome and mitogenome of *D. yangii.* Furthermore, we conducted analyses, including codon analysis, Ka/Ks analysis, repeat sequence analysis, and an examination of the intracellular gene transfer (IGT) events as well as phylogenetic relationships among *D. yangii* and 32 other species using the mitogenome. We believe that our results can serve as valuable molecular resources for future research on the genetic variation and systematic evolution of orchids.

## 2. Results

### 2.1. Characterization and Comparative Analysis of Plastomes and Mitogenomes

**Figure 1:**
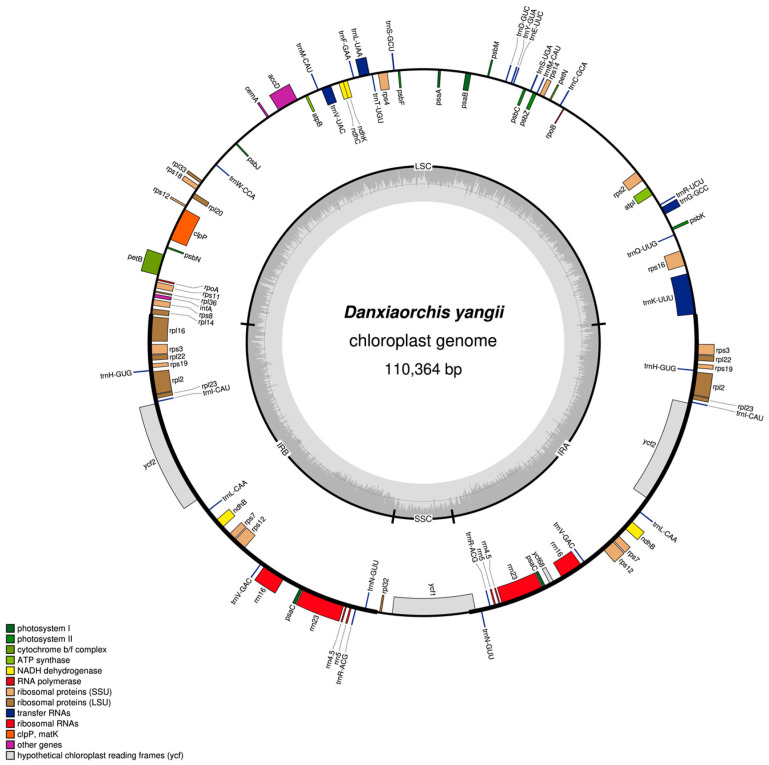
Plastid genome map of *D. yangii*. The darker gray in the inner circle corresponds to the GC content. The IRa and IRb (two inverted repeating regions), LSC (large single-copy region), and SSC (small single-copy region) are indicated outside of the GC content.

The complete plastome size of *D. yangii* exhibits a typical quadripartite structure and is 110,364 bp in length (Figure 1), with a total GC content of 36.60%. It contains a pair of inverted repeats (IRs; 26,450 bp each) that separate the large single-copy (LSC; 51,523 bp) region from the small single-copy (SSC; 5941 bp) region. In total, 94 genes were annotated, comprising 57 protein-coding genes (PCGs), 29 transfer RNAs (tRNAs), and 8 ribosomal RNAs (rRNAs). Plastid genes encoding subunits of the NAD(P)H dehydrogenase complex (*ndh* genes) have mostly been partially lost or pseudogenized in *D. yangii*.

**Figure 2:**
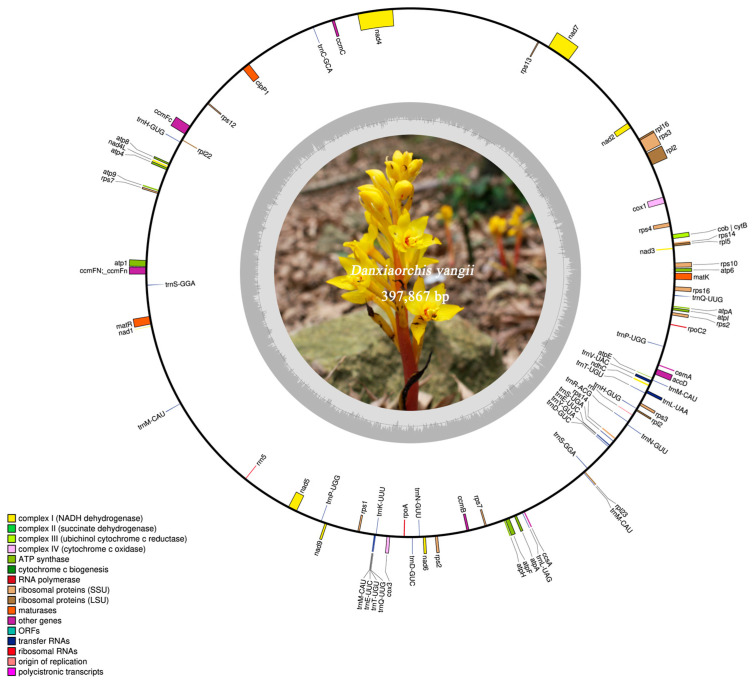
Mitochondrial annotation map of *D. yangii*. The dark gray region represents the GC content, while the light gray region represents the AT content.

Through de novo assembly, the mitogenome size of *D. yangii* was determined to be 397,867 bp, consisting of 41 contigs, and a circular structure was manually depicted (Table 1, Figure 2). The GC content was measured to be 42.70%. In total, 55 mitochondrial genes were annotated, comprising 37 coding sequences (CDSs), 16 tRNAs, and 2 rRNAs. The lengths of tRNAs ranged from 66 bp (*trnP*-*UGG*) to 94 bp (*trnS*-*UGA*). The PCGs included ATP synthase genes (*atp1*, *atp4*, *atp6*, *atp8*, *atp9*, *atpA*, *atpE*, *atpF*, *atpH*, and *atpI*), nine NADH dehydrogenase genes (*nad1*, *nad2*, *nad3*, *nad4*, *nad4L*, *nad5*, *nad6*, *nad7*, and *nad9*), five cytochrome c biogenesis genes (*ccmB*, *ccmC*, *ccmFc*, and *ccmFN*), one maturase gene (*matR*), two cytochrome c oxidase genes (*cox1* and *cox3*), and one ubichinol cytochrome c reductase gene (*cob*).

### 2.2. Codon Preference Analysis

**Figure 3:**
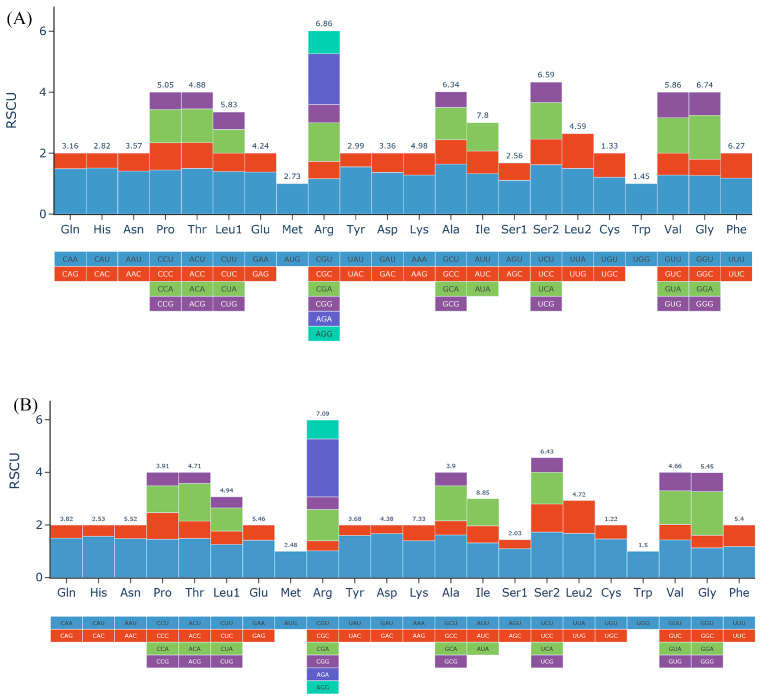
(**A**) Relative synonymous codon usage (RSCU) in *D. yangii*’s mitogenome. (**B**) Relative synonymous codon usage (RSCU) in *D. yangii*’s plastome.

We carefully estimated codon usage frequency associated with all protein-coding sequences in the *Danxiaorchis* mitogenome and plastome (Supplementary Table S1). As depicted in Figure 3A, 22 amino acids and 61 codons were identified from *D. yangii*’s mitogenome (Supplementary Table S1). Of these, 25 codons had relative synonymous codon usage (RSCU) values greater than 1, while 31 codons had values less than 1. Notably, the codons ACC (Threonine), AUG (Methionine), GUA (Valine), UCC (Serine), and UGG (Tryptophan) all had an RSCU of 1. The codon AGA (Arginine) had the highest RSCU value of 1.83, followed by CUU (Leucine) at 1.46, while CGU (Arginine) had the lowest at 0.63. Among the six codons for Arg, AGA and AGG (1.24) were utilized most frequently. For Leu, CUU (1.46) exhibited the highest usage for Leu, and UCU had the highest RSCU for Serine (1.44). In light of RSCU analysis on *D. yangii*’s plastome, the results also showed that 36 codons exhibited RSCU values greater than 1 and 28 codons exhibited less than 1 (Figure 3B, Supplementary Table S1). Analysis of the relative synonymous codon usage (RSCU) indicated that UCU and AGA had the highest CUB, with average values of 1.77 and 2.105, respectively, whereas AGC and GAC had the lowest CUB, with average values of 0.305 and 0.33, respectively. All preferred RSCU (RSCU > 1) ending with an A or a U, except for UUG, UCC, and UAG.

### 2.3. Repeat Sequence

**Figure 4:**
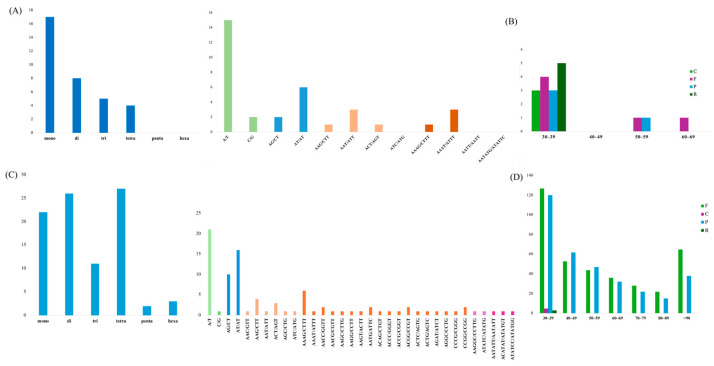
Comparative analysis of repeat sequences between the *D. yangii* plastome and mitogenome. (**A**) SSR distribution in the plastome. (**B**) Long repeats in the plastome. (**C**) SSR distribution in the mitogenome. (**D**) Long repeats in the mitogenome.

Repetitive sequences consist of simple sequence repeats (SSRs), tandem repeats, and dispersed repeat sequences. A total of 34 SSRs were identified in *D. yangii*’s plastome, with mononucleotide SSRs being the most abundant, accounting for 50% (17 repeats). Pentanucleotide and hexanucleotide were absent (Figure 4A,B, Supplementary Table S2). Among the repeat motifs, the A/T (15) was the most frequently observed, followed by six AT/AT motifs. Additionally, 18 long repeats were identified in the plastome, categorized into three length ranges: 30–39, 50–59, and 60–69 bp. The 30–39 range was the most abundant, with 15 repeats, followed by 50–59 bp (2 repeats) and 60–69 bp (1 repeat).

In the *D. yangii*’s mitogenome, a total of 91 SSRs were discovered, including 22 (24.17%) monomers, 26 (28.57%) dimers, 11 (12.09%) trimers, 27 (29.67%) tetramers, 2 (2.20%) pentamers, and 3 (3.30%) hexamers (Figure 4C,D, Supplementary Table S3). Analysis of the SSR units revealed that G/C (1) occupied only 4.55% of the monomers, whereas A/T (21) monomers accounted for 95.45% (Figure 4C). As shown in Figure 4D, a total of 719 long repeats were identified in the mitogenome. Notably, there were only five and three repeats of the complement (C) and reverse (R) types, respectively. It is worth noting that types C and R were identified exclusively in the 30–39 bp range. Long repeats were distributed as follows: 115 repeats in the 40–49 bp range, 91 in the 50–59 bp range, and 103 in the >90 bp range, while in the 60–69, 70–79, and 80–89 bp ranges, there were 68, 50, and 37 repeats, respectively.

### 2.4. Gene Transfer Between Organelle Genomes

**Figure 5:**
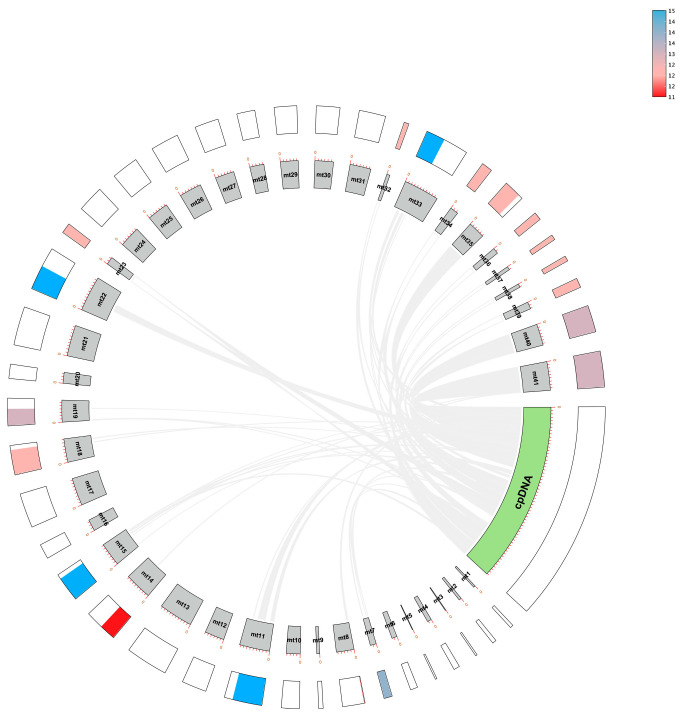
Gene transfer between organelle genomes. Gene transfer between the plastome and mitogenome of *D. yangii*. The outer circle represents the names and lengths of mitochondrial contigs and plastids. The middle circle indicates gene density, while the innermost circle displays the distribution of genes.

To identify horizontal gene transfer between organelles in *D. yangii*, we performed collinearity analysis between its mitochondrial genome (397,867 bp) and chloroplast genome (110,364 bp), identifying 64 potential collinear fragments ranging from 65 to 17,455 bp (Figure 5). Within these homologous regions, certain plastid genes were identified, including two tRNA genes (*trnD*-*GUC* and *trnN-GUU*), *accD*, *clpP*, *rpl2*, *rps2*, *matk*, *petA*, and photosystem genes (*psaA*, *psaB*, *psbJ*, *psbK*, *psbM*, and *psbZ*) (Supplementary Table S4). Additionally, it was observed that the majority of these regions were rRNA regions, while the coding gene regions were too short for further comparison.

### 2.5. Genome Synteny Evolution and Phylogenetic Analysis

**Figure 6:**
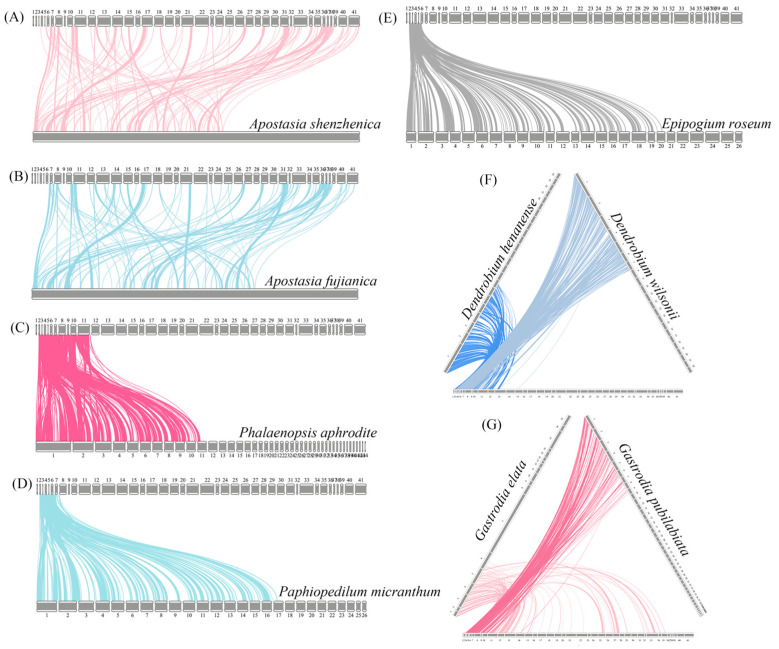
Collinearity plots of the mitogenomes among *D. yangii* against nine other orchids, including *A. shenzhenica* (**A**), *A. fujianica* (**B**), *Pha. aphrodite* (**C**), *Pap. micranthum* (**D**), *E. roseum* (**E**), *Dendrobium* species (**F**), and *Gastrodia* species (**G**). The boxes in each row represent the mitogenomes, and the lines in the middle represent the homologous regions.

The collinearity analysis of mitochondrial genomes between D. yangii and nine other orchids, including *Apostasia shenzhenica*, *A. fujianica*, *Dendrobium wilsonii*, *Den. henanense*, *Epipogium roseum*, *Gastrodia pubilabiata*, *G. elata*, *Phalaenopsis aphrodite*, and *Paphiopedilum micranthum*, was conducted using BLASTN to identify conserved homologous sequences (Supplementary Table S5). Sequences that exceeded 500 bp and met a size threshold of 0.5 kb were considered conserved collinear blocks and included in the analysis. Totals of 149, 141, 311, 212, 204, 240, 259, 223, and 231 homologous collinear blocks were identified for *D. yangii and A. shenzhenica*, *A. fujianica*, *Pha. aphrodite*, *Pap. micranthum*, *E. roseum*, *Den. henanense*, *Den. wilsonii*, *G. elata*, and *G. pubilabiata*, respectively (Figure 6). Synteny analysis revealed extensive interspecies large homologous regions undergoing structural recombination, as evidenced by numerous cross-linking patterns.

**Figure 7:**
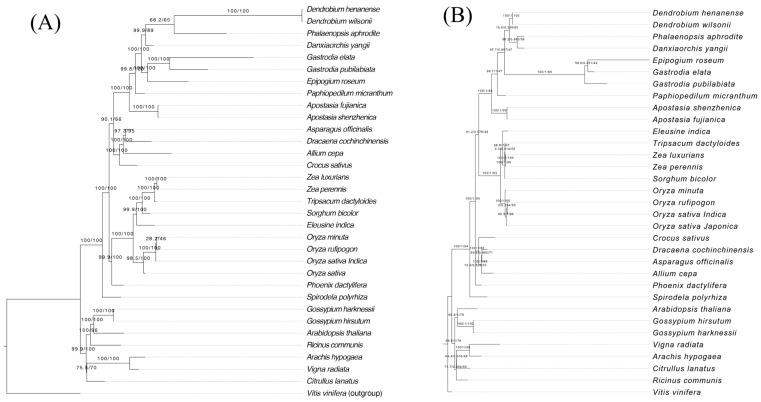
Phylogenetic analysis. (**A**) A maximum likelihood (ML) phylogenetic tree of 33 mitogenomes based on 24 PCGs. (**B**) A maximum likelihood (ML) phylogenetic tree based on 83 PCGs from 33 plastomes. The numbers near the nodes are bootstrap percentages.

To determine the phylogenetic position of *D. yangii*, we retrieved mitochondrial genome sequences of 32 angiosperms from GenBank (Supplementary Table S5). The mitochondrial phylogenetic analysis was based on 24 conserved mitochondrial PCGs of 33 species, using *Vitis vinifera* as the outgroup (Figure 7A), indicating that *D. yangii* is grouped with a clade that consisted of *Pha. aphrodite* and *Dendrobium* species. Compared to mitochondrial phylogenetic analysis, plastid phylogenetic relationships based on 83 plastid PCGs from these 33 species showed that *D. yangii* forms a sister group with *Pha. aphrodite*, further representing a sister group to the *Dendrobium* clade (Figure 7B). Nevertheless, phylogenetic analyses based on both mitogenomes or plastomes revealed that *Pha. aphrodite* is the closest relative to *D. yangii*, which aligns with the finding that *D. yangii* shares the most collinear blocks with *Pha. aphrodite* (Figure 6C).

### 2.6. Selective Pressure Analysis

To explore the selection pressure on the plastid and mitochondrial protein-coding genes, the non-synonymous (dN) and synonymous (dS) substitution rates were separately calculated for *D. yangii* against nine other orchids—*A. shenzhenica*, *A. fujianica*, *Den. wilsonii*, *Den. henanense*, *E. roseum*, *G. pubilabiata*, *G. elata*, *Pha. aphrodite*, and *Pap. micranthum* (Supplementary Tables S6 and S7).

**Figure 8:**
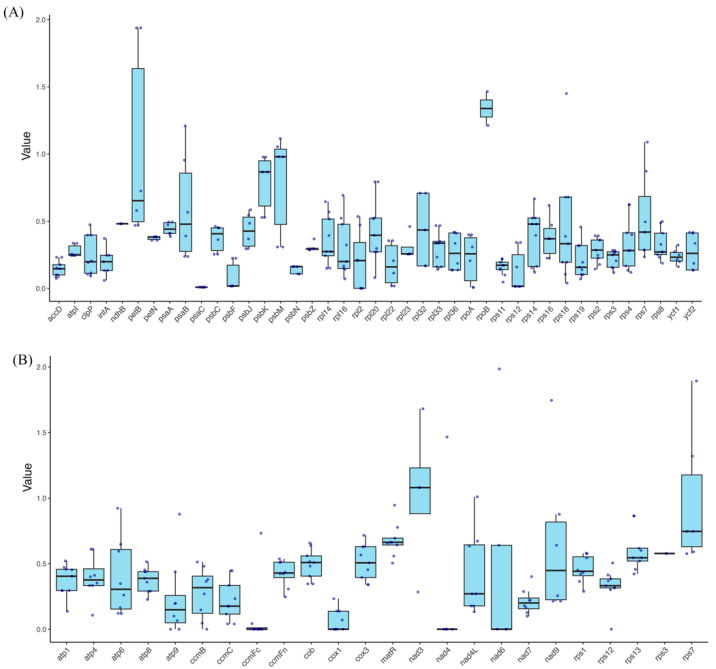
Selective pressure (ω = dN/dS) of PCGs in *D. yangii*’s plastome (**A**) and mitogenome (**B**) compared to the nine other orchids.

As shown in Figure 8A and Table S6, the selective pressure analysis of these orchids’ plastomes reveals significant variation in ω values across different genes. For instance, the *petB* gene shows exceptionally high ω values in *Den. henanense* and *Den. wilsonii* (1.9391), indicating the strong positive selection within the *Dendrobium* lineage. On the other hand, genes like *accD* and *psaC* have consistent ω values across species, suggesting that they are likely subject to stabilizing selection throughout the Orchidaceae family. Overall, most genes (e.g., *psaC* and *rps12*) have ω values significantly below 1, implying strong purifying selection across most species to maintain functional stability. However, the ω value of *rps18* in *Pha. aphrodite* reaches 1.4503, suggesting positive selection, as does *rpoB* in both *Pap. micranthum* and *Pha. aphrodite* (ω = 1.2124 and 1.4653, respectively).

As shown in Figure 8B and Table S7, genes related to ATP synthesis, including *atp1*, *atp4*, *atp6*, *atp8*, and *atp9*, as well as genes involved in photosynthesis and respiration (e.g., *ccmB*, *ccmC*, and *cox1*), generally exhibit low ω values. This suggests these genes are under conserved selective pressure across most orchid species, maintaining their stable functions. Additionally, the ω values of genes such as *nad3*, *nad4*, and *nad7* vary widely among species. For example, *nad3* in *Den. wilsonii* has a high ω value of 1.6812, suggesting positive selection or adaptive changes in this species. In contrast, the ω values of *rps12* and *rps13* are predominantly less than 1, indicating that these genes are primarily subject to purifying selection, except in cases with specific mutations. Some genes, like *nad4L*, *nad6*, and *nad9*, show higher ω values (e.g., *nad4L* in *G. elata* = 1.0103), which may suggest that these genes have undergone positive selection or adaptive evolution in response to particular environmental pressures.

## 3. Discussion

### 3.1. Characterization of the D. yangii Organelle Genomes

In most angiosperms, plastid genomes are inherited maternally and show minimal recombination, maintaining a highly conserved structure among closely related species [24]. Similar to other angiosperms, the *Danxiaorchis* plastomes exhibit a typical quadripartite structure, consisting of one LSC, one SSC, and two IR regions. Generally, land plants have chloroplast (cp) genomes ranging from 120 to 160 kb in size; those with differences in cp genome size are mostly influenced by the expansion/contraction of IR regions due to changes in the amount of repeated DNA and/or sequence complexity [25]. The IR regions of the newly reported cp genome (26,450 bp) are significantly larger than those of two published *Danxiaorchis* species (13,762 bp and 11,951 bp), while the SSC region is the smallest (5940 bp) (Table 2). This is evident in the cp genome size of *D. yangii* (110,364 bp), which is larger than that of the other two *Danxiaorchis* plastomes (87,931 bp of *D. singchiana*; 85,273 bp of *D. mangdangshanensis*), due to the expansion of IR length. Additionally, the LSC length of *D. yangii* (51,523 bp) is slightly larger than that of *D. singchiana* (42,575 bp) and *D. mangdangshanensis* (42,605 bp). This indicates that the expansion of IR into SSC region has contributed to a reduction in the SSC region of *D. yangii*, a phenomenon also reported in *Cypripedium* and *Corydalis* species [26,27].

Previous studies have explored the complex structures and varying genome sizes of plant mitogenomes [3]. According to the literature, the mtDNA length of orchids generally ranges from 414,552 bp (*E. roseum*) and 1,340,105 bp (*G. elata*). Our study is the first to report the *D. yangii* mitogenome, which consists of 41 contigs with a total length of 397,867 bp, has the highest reduced length observed in orchids to date. A total of 55 genes were successfully annotated, including 37 protein-coding genes (PCGs), 16 tRNA genes, and 2 rRNA genes. The rapid acquisition or loss of chromosomes was postulated as a pivotal evolutionary process that explains these observed disparities. However, some PCGs were incomplete or showed pseudogenization.

### 3.2. Repeat Sequences

Repeat sequences are potentially important markers for population and evolutionary studies. Mitochondrial repeat sequences are essential for intermolecular recombination, as they can contribute to extreme mitogenome sizes and structural differences [28,29]. In this study, we identified long repeats and simple sequence repeats (SSRs) in the *D. yangii* mitogenome. We found that long repeats of 30–39 are the most abundant (255), similar to the situation observed in *A. fujianica* (250–300) [30]. In SSR analysis, A/T repeats were the most prevalent among all SSRs (91), a pattern also noted in other orchids, such as *E. roseum* [8] and *A. fujianica* [30]. SSRs offer advantages such as codominance, high reproducibility, and the requirement for a small amount of DNA template [31]. These features facilitate their application in various contexts, including DNA fingerprinting, gene mapping, and marker-assisted breeding [32,33,34].

### 3.3. DNA Fragment Transfer Events

Both mitochondria and chloroplasts were once independent prokaryotes. Over time, cp genomes became progressively smaller, while mitogenomes gradually expanded due to frequent exchanges with nuclear and chloroplast DNA [35]. In plants, the mitogenome is considerably larger than the cp genome [36]. In the present study, the mitogenome (397,867 bp) is nearly 3.5 times larger than the cp genome (113,034 bp), the finding that aligns with previous research highlighting substantial similarities between mitochondrial and cp genomes [37]. The presence of plastome-derived sequences in plant mitogenomes can be attributed to inter-organelle gene transfers (IGTs) and horizontal gene transfers (HGTs). For instance, in *Saposhnikovia divaricate*, 10 groups of mitogenome sequences, representing 6.1% of the mitogenome, were similar to its cp genome sequences. Herein, we identified 64 short fragments potentially transferred from the cp genome to the mitogenome, including 16 potential genes (*trnD-GUC*, *trnN-GUU*, *accD*, *clpP*, *rpl2*, *rps2*, *matk*, *petA*, *psaA*, *psaB*, *psbJ*, *psbK*, *psbM*, and *psbZ*).

### 3.4. Genome Synteny Evolution and Phylogenetic Analyses

The mitochondrial or chloroplast sequences have been widely used to infer phylogenetic relationships within Orchidaceae [29]. In this study, we investigated the individual phylogenetic analysis of *D. yangii* based on 24 mito-PCGs or 83 cp-PCGs from 33 species. The mitochondria phylogenetic relationships indicate that *D. yangii* is grouped as follows: *D. yangii* + ((*Den.wilsonii* + *Den. henanense*) + *Pha. aphrodite*)). As molecular taxonomy studies of Orchidaceae continue to increase, particularly with the acquisition of new mitochondrial DNA (mtDNA), the phylogenetic relationships observed in this study may evolve. Therefore, it is essential to expand taxon sampling to reconstruct more reliable phylogenies for Orchidaceae, especially in biodiversity-rich regions. The chloroplast phylogenetic relationships indicate that *D. yangii*, sister to *Pha. aphrodite*, is grouped to the *Dendrobium* clade.

### 3.5. Gene Selective Pressure Analysis

Based on the selective pressure analysis, most genes in the mitochondrial and plastid genomes of D. yangii show ω values close to or below 1, indicating that these genes are under purifying selection, maintaining functional stability. For example, ATP synthesis-related genes (*atp1*, *at sp4*, *atp6*, *atp8*, and *atp9*) and genes involved in photosynthesis and the respiratory chain (*ccmB*, *ccmC*, *ccmFc*, *ccmFn*, *cob*, and *cox1*) generally exhibit low ω values, suggesting that conserved selection should be carried out to maintain essential functions critical for energy metabolism and photosynthesis. Additionally, chloroplast genes like *psaC* and *rps12* also show low ω values, further supporting the idea that these genes are under purifying selection to preserve their functions. The petB gene exhibits a high ω value in Den. *henanense* and *Den. wilsonii* (1.9391), suggesting that this gene may have undergone positive selection in these *Dendrobium* lineage. Environmental factors, such as light intensity and temperature, may have driven this positive selection, enhancing the photosynthetic efficiency of these plants in high-light environments [38,39]. This adaptive evolution likely reflects the influence of specific environmental pressures, allowing *Dendrobium* species to optimize their photosynthetic performance in habitats with intense light, demonstrating how environmental conditions can shape the evolution of key genes like *petB*. We found that rpoB exists only in *Pap. micranthum* and *Pha. aphrodite*, with ω values greater than 1 (1.2124 and 1.4653, respectively). Overall, while most mitochondrial and plastid genes in *D. yangii* are under purifying selection, ensuring functional stability, certain genes may have undergone positive selection or adaptive evolution, reflecting the species’ adaptation to specific ecological environments. The variation in selective pressures provides valuable molecular evidence for understanding how *D. yangii* adapts to its unique habitat.

## 4. Materials and Methods

### 4.1. Plant Material and Sequencing

Fresh aboveground tissue of *D. yangii* was collected from Ji’an City, Jiangxi Province, China (26°66′10.18″ N, 115°41′8.78″ E). Total genomic DNA was extracted from fresh individuals using the Plant Genomic DNA Kit (Tiangen, Beijing). The qualified libraries were sequenced on the MGISEQ-2000 platform. After removing adapter sequences with NGSToolkit v.2.3.3, the raw reads were subjected to de novo assembly using GetOrganelle v1.7.6.1 [40]. We used SOAPnuke1.5.6 (https://github.com/BGI-flexlab/SOAPnuke, accessed on 16 October 2023) to eliminate adapters and low-quality data (bases with quality value Q ≤ 20 that account for more than 10% of the total read and a proportion of ‘N’ greater than 1%), following these specific parameters: -n 0.01 -l 20 -q 0.1 -i -Q 2 -G -M 2 -A 0.5 -d.

### 4.2. Assembly, Annotation, and Condon Usage Analysis

We used SPAdes v3.10.1 [41] for the de novo assembly of the *D. yangii* mitogenome. Multiple SPAdes runs were conducted with different k-mer values (k = 77, 101, and 127), and QUAST [42] was utilized to evaluate and select 127 as the optimal k-mer number for multiple assembly processes. Ultimately, we identified only one candidate mitochondrial scaffold, which can be mapped as a circular molecule with a pair of direct repeats at its both ends. Sanger sequencing was used to confirmed the connector and filled the seven remaining gaps in this scaffold. For gene annotation, the coding and rRNA genes were annotated in Geneious Prime [43], using published orchid mitogenomes as references. Manual verification and correction ensured the accuracy of the annotation results. OGDRAW version 1.3.1 [44] was used for visualization, illustrating the types and quantities of mitochondrial genes, as well as the GC content of *D. yangii* mitogenome. Relative synonymous codon usage (RSCU) was analyzed using DAMBE 7 [45].

### 4.3. Analysis of Repeat Structure

MISAv2.1 [46] (https://webblast.ipk-gatersleben.de/misa/, accessed on 1 September 2022) was utilized to assess the simple sequence repeats (SSRs) of the *D. yangii* mitogenome, with the motif size ranging from one to six nucleotides and thresholds set at 10, 5, 4, 3, 3, and 3, respectively. The Tandem Repeats Finder v4.09 program [47] (http://tandem.bu.edu/trf/trf.submit.options.html, accessed on 1 September 2022) was used with default parameters to find tandem repeats with a >6 bp repeat unit. Additionally, dispersed repeats, including forward, reverse, palindromic, and complementary repeats, were discovered using the REPuter web server (https://bibiserv.cebitec.uni-bielefeld.de/reputer, accessed on 5 September 2022) with the following parameters: “Hamming Distance 3, Maximum Computed Repeats 5000, Minimum Repeat Size 30” [48].

### 4.4. Gene Transfer, Synteny Anlysis, and Phylogenetic Analysis

BLASTN [49] was employed to analyze sequence similarity between the cp and the mitogenome to detect transferred DNA fragments, with an e-value cut-off of 1 × 10^−5^. The Circos module implemented in TBtools v1.105 was used to visualize the results [50]. Homologous sequences longer than 500 bp were retained to construct multiple synteny plots as conserved collinear blocks and pairwise comparisons of individual mitochondrial genomes were conducted using BLASTN with the parameters set to an e-value of ≤1 × 10^−10^ and a matching rate of ≥80% [51]. The R program (version 4.3.1) was employed to generate multiple synteny plots for nine species [52].

To construct a maximum likelihood (ML) phylogenetic tree, mitochondrial genome data for 33 species were downloaded from the National Center for Biotechnology Information (NCBI, https://www.ncbi.nlm.nih.gov/, accessed on 20 February 2024). The 24 PCGs (*atp1*, *atp4*, *atp6*, *atp8*, *atp9*, *ccmB*, *ccmC*, *ccmFc*, *ccmFn*, *cob*, *cox1*, *cox2*, *cox3*, *matR*, *nad1*, *nad2*, *nad3*, *nad4*, *nad4L*, *nad5*, *nad6*, *nad7*, *nad9*, *rps1*, *rps3*, *rps7*, *rps12*, and *rps13*) were extracted from the mitogenomes using Geneious Prime v2023.2.1. The extracted 24 FASTA files were then aligned using MACSE module in PhyloSuite v1.2.3 [53], followed by sequence concatenation. The ModelFinder module in PhyloSuite v1.2.3 was used to generate partition mode. Finally, the ML phylogenetic tree was constructed using IQ-TREE with 1000 bootstrap replicates, and the tree was visualized using FigTree v1.4.4. The phylogenetic tree of 83 PCGs from 33 species was constructed using the same method. We estimated the non-synonymous (dN) and synonymous (dS) substitution rates, as well as the dN/dS (ω) value to detect the selective pressure on the cp or mito genes for *D. yangii* against nine other orchids. The relative values of dN and dS in each CDS were calculated using pairwise model in the PAML v.4.8 software package [54,55].

## 5. Conclusions

In this study, we present the first complete plastome (110,364 bp; 64 PCGs) and mitogenome (397,867 bp; 55 PCGs) of the fully mycoheterotrophic orchid *Danxiaorchis yangii*. By comparing the mitogenome and cp genome sequences, we detected 64 short fragments potentially transferred from the cp genome to the mitochondrial genome, highlighting the dynamic nature of potentially genetic exchange between organelles, which warrants further experiments to verify this interesting phenomenon. Furthermore, we conducted an extensive analysis of *D. yangii* mitogenome focusing on codon usage patterns, sequence repeats, and selection pressures. Mitochondrial and plastome phylogenetic analyses both place *D. yangii* in a clade with *Pha*. *aphrodite* and *Dendrobium* lineage, with *Pha. aphrodite* as its closest relative, consistent with the finding that *D. yangii* shares the most collinear blocks with *Pha. aphrodite.* Additionally, gene selective pressures analysis revealed that most of the mitochondrial and plastid genes in *D. yangii* exhibited low ω values, indicating that these genes are under purifying selection, which is essential for maintaining stable functional roles, such as energy metabolism. However, some genes, including *petB* in *Dendrobium* species and *nad3* in *Den. wilsonii*, showed higher ω values, suggesting potential positive selection or adaptive evolution in specific species. This study offers insights into the evolutionary adaptations of this unique orchid and broadens our understanding of organelle genome dynamics in non-photosynthetic mycoheterotrophic plants, paving the way for future research on their evolution and ecology.

## Figures and Tables

**Table 1:** Characteristics of the complete mitogenome of *D. yangii*. ^#^ Genes were incomplete or showed pseudogenization.

Contig	Length (bp)	GC Content (%)	Genes
mt1	1115	42.2	*atp6*, *nad7*
mt2	1553	45.8	*rps10*
mt3	379	40.1	*cob*
mt4	2543	46.9	*cox2*
mt5	373	45.3	*nad3*, *rps12*
mt6	3191	43.4	*rpl5*, *rps14*
mt7	3742	41.1	*rps4*
mt8	10,919	43.3	*cox1*
mt9	1754	42.1	/
mt10	8534	47.0	/
mt11	18,663	41.0	/
mt12	12,075	48.7	*nad7*
mt13	20,870	45.2	*rps13*
mt14	16,689	45.3	/
mt15	14,399	48.7	*nad4*
mt16	7046	45.8	/
mt17	16,880	42.8	/
mt18	14,342	41.5	*trnQ-UUG*
mt19	13,337	43.7	*rpl22*, *ccmFc*, *trnC-GCA^#^*
mt20	6655	43.6	*atp4*, *nad4L*
mt21	18,953	44.7	*trnK-UUU*
mt22	21,634	43.0	*atp9*, *nad1^#^*, *ccmFn*, *mat-R*, *trnS-GGA*
mt23	4746	48.7	/
mt24	12,812	46.3	/
mt25	13,033	43.6	/
mt26	13,736	46.4	/
mt27	11,662	44.6	*nad5*
mt28	8694	42.8	*nad9*
mt29	10,921	39.6	*atp1*, *atp8*, *rps1*, *trnH-GUG*
mt30	11,402	42.0	*cox3*, *nad6*, *trnD-GUC*
mt31	12,982	43.6	*rps2*, *ccmB*
mt32	1979	39.0	*rps7*
mt33	21,895	42.6	*nad2*, *trnL-UAG*
mt34	5046	38.3	*rpl23*, *ccmC*
mt35	10,405	33.8	*trnE-UUC*, *trnY-GUA*, *trnS-UGA*
mt36	3560	40.5	/
mt37	2171	44.5	*trnR-ACG*, *trnN-GUU*
mt38	2205	55.7	/
mt39	4356	34.5	*rps3.rpl2*, *rpl16*, *trnH-GUG*
mt40	13,064	31.9	*rps4*, *trnT-UGU*, *trnL-UAA*, *trnV-UAC*
mt41	17,561	31.1	*rps2*, *rps16*, *trnQ-UUG*

**Table 2:** General characteristics of the chloroplast genomes of three *Danxiaorchis* species.

Species	GenBank No.	Length (bp)	LSC (bp)	SSC (bp)	IR (bp)	GC %	Coding Genes	tRNAs	rRNAs
*D. yangii*	OR569720	110,364	51,524	5940	26,450	36.60	57	29	8
*D. singchiana*	NC_048523	87,931	42,575	17,831	13,762	34.55	35	28	4
*D. mangdangshanensis*	OP122564	85,273	42,605	18,766	11,951	34.00	32	20	4

## Data Availability

Accession numbers for the newly completed plastid and mitochondrial genomes are OR569720 and PQ306624, respectively. The sequence data utilized in this study can be found in Supplementary Materials.

## Supplementary Materials

The following supporting information can be downloaded at: https://www.mdpi.com/article/10.3390/ijms26020562/s1.
